# Evaluation of serum sphingolipids and the influence of genetic risk factors in age-related macular degeneration

**DOI:** 10.1371/journal.pone.0200739

**Published:** 2018-08-02

**Authors:** Luciana M. Pujol-Lereis, Gerhard Liebisch, Tina Schick, Yuchen Lin, Felix Grassmann, Koji Uchida, Peter F. Zipfel, Sascha Fauser, Christine Skerka, Bernhard H. F. Weber

**Affiliations:** 1 Institute of Human Genetics, University of Regensburg, Regensburg, Germany; 2 Institute of Clinical Chemistry and Laboratory Medicine, University of Regensburg, Regensburg, Germany; 3 Department of Ophthalmology, University Hospital of Cologne, Cologne, Germany; 4 Department of Infection Biology, Leibniz Institute for Natural Product Research and Infection Biology, Jena, Germany; 5 Graduate School of Agricultural and Life Sciences, The University of Tokyo, Tokyo, Japan; 6 F. Hoffmann—La Roche, Basel, Switzerland; University of Manchester, UNITED KINGDOM

## Abstract

Sphingolipids are bioactive molecules associated with oxidative stress, inflammation, and neurodegenerative diseases, but poorly studied in the context of age-related macular degeneration (AMD), a prevalent sight-threatening disease of the ageing retina. Here, we found higher serum levels of hexosylceramide (HexCer) d18:1/16:0 in patients with choroidal neovascularization (CNV) and geographic atrophy (GA), two manifestations of late stage AMD, and higher ceramide (Cer) d18:1/16:0 levels in GA patients. A sensitivity analysis of genetic variants known to be associated with late stage AMD showed that rs1061170 (p.Y402H) in the complement factor H (*CFH*) gene influences the association of Cer d18:1/16:0 with GA. To understand the possible influence of this genetic variant on ceramide levels, we established a cell-based assay to test the modulation of genes in the ceramide metabolism by factor H-like protein 1 (FHL-1), an alternative splicing variant of CFH that also harbors the 402 residue. We first showed that malondialdehyde-acetaldehyde adducts, an oxidation product commonly found in AMD retinas, induces an increase in ceramide levels in WERI-Rb1 cells in accordance with an increased expression of ceramide synthesis genes. Then, we observed that cells exposed to the non-risk FHL-1:Y402, but not the risk associated variant FHL-1:H402 or full-length CFH, downregulated ceramide synthase 2 and ceramide glucosyltransferase gene expression. Together, our findings show that serum ceramide and hexosylceramide species are altered in AMD patients and that ceramide levels may be influenced by AMD associated risk variants.

## Introduction

Age-related macular degeneration (AMD) is a multifactorial progressive disease of the central retina and the leading cause of blindness in developed countries in people over 60 years of age [1]. There are two forms of late stage AMD including the non-exudative form, also known as geographic atrophy (GA), and the exudative form. Pathological changes leading to GA are characterized by drusen, which are deposits of lipids and proteins, loss of retinal pigment epithelium (RPE), and gradual degeneration of the outer layers of the neurosensory retina, together with atrophy of the choriocapillaris [2]. The hallmark of exudative AMD is choroidal neovascularization (CNV), which involves mainly the formation of new blood vessels typically growing from the choroid through Bruch’s membrane and the RPE.

Strong genetic risk factors for AMD were found in the complement factor H (*CFH*) gene locus [3–5], and the age-related maculopathy susceptibility 2/ HtrA serine peptidase 1 (*ARMS2/HTRA1*) interval [6–8]. Although with lower effect sizes, several lipid pathway genes have also been significantly associated with AMD risk, including apolipoprotein E (*APOE*), cholesteryl ester transfer protein (*CETP*), hepatic triglyceride lipase (*LIPC*), ATP-binding cassette transporter A-1 (*ABCA1*), *ABCA7*, lipoprotein lipase (*LPL*), and fatty acid desaturase 1 (*FADS1*) [9–11]. The proteins encoded by these genes are involved in lipid transport between lipoproteins and from lipoproteins to tissues/cells, and vice-versa. The fact that lipoproteins and cholesterol are major constituents of drusen has motivated previous studies to investigate into circulatory lipid levels of AMD patients. Contradictory results were reported regarding the association of HDL-cholesterol, LDL-cholesterol, total cholesterol and triglycerides levels with AMD status, and some studies even failed to find an association altogether [12–28]. Regarding circulating fatty acids, higher levels of total n3 fatty acids, α-linolenic acid (18:3-n3) and long chain n3 fatty acids were associated with a reduced risk for late AMD, with no significant association of docosahexaenoic acid (22:6-n3) or eicosapentanoic acid (20:5-n3) [29]. A subsequent study showed an association of circulating eicosapentanoic acid with lower risk for CNV [30]. Besides unesterified cholesterol and triglycerides, other lipid classes present in lipoproteins are phospholipids, cholesteryl esters, and sphingolipids such as sphingomyelins (SM) and ceramides [31].

Many sphingolipids are bioactive molecules and participate in signaling pathways involved in apoptosis, autophagy, inflammation, and stress response [32–34]. Ceramide can activate inflammatory pathways via induction of transcription factor families such as nuclear factor-kB (NF-kB) [35], and CCAAT/enhancer binding proteins (c/EBP) [36], which in turn induce genes encoding cytokines, chemokines and pro-inflammatory enzymes. It was also demonstrated that exogenous ceramides can induce oxidative stress and apoptosis in human RPE cells, an effect that can partially be prevented by antioxidants [37]. In the context of neurological disorders, a pioneering study showed that serum SM and ceramides were suited to predict cognitive impairment, and ceramide (Cer) d18:1/16:0 and stearoyl ceramides predicted impairment on delayed and immediate memory recall, and psychomotor speed [38]. Subsequently, Cer d18:1/16:0, Cer d18:1/24:0 and lactosylceramide were associated with a higher risk of Alzheimer’s disease (AD) [39]. Also, several ceramide and hexosylceramide species were found altered in plasma of patients with sporadic Parkinson’s disease and cognitive impairment compared to controls [40]. In a non-targeted lipidomics study of plasma, lower levels of eight SM species containing long aliphatic chains, and higher levels of two ceramide species (Cer d18:1/16:0 and Cer d18:1/21:0) were observed in AD patients compared to controls [41]. In addition, plasma SM ratios were found to be associated with depression and anxiety symptoms [42].

There is limited clinical research that evaluated the role of sphingolipids in the pathogenesis of AMD. A recent case-control study examined plasmatic gangliosides, that are synthesized by sequential glycosylation of ceramides, but failed to find significant differences in the levels of these glycosphingolipids between controls, GA and CNV patients [43]. In the present study, serum levels of ceramides and SM from late stage AMD patients and healthy age-matched controls were quantified by electrospray ionization tandem mass spectrometry (ESI-MS/MS). Our main goal was to determine if serum sphingolipid levels were altered in late AMD, and to examine the influence of genetic variants previously associated with the disease. We found that specific ceramide species are elevated in patients with late stage AMD compared to controls. Interestingly, genetic variant rs1061170 (p.Y402H) in *CFH* and variant rs10490924 in *ARMS2* tend to increase the association between Cer d18:1/16:0 and disease. Given these interesting findings, we established a cell-based assay to test the hypothesis that variant rs1061170 in *CFH* may be involved in regulating ceramide metabolism.

## Materials and methods

### Study population

A total of 373 participants above the age of 55 years from Cologne, Germany, were included in this study (The European Genetic Database, EUGENDA, www.eugenda.org). The study was performed in accordance with the tenets of the Declaration of Helsinki and the Medical Research Involving Human Subjects Act (WMO), and was approved by the local ethics committee of the University Hospital in Cologne. Informed written consent was obtained from all participants.

AMD and control status were assigned by multimodal image grading that included stereo fundus photographs, fluorescein angiograms, and spectral domain optical coherence tomograms. The grading was performed according to the standard protocol of the Cologne Image Reading Center (CIRCL) by certified graders. The classification of AMD and grading procedures were performed as described previously [44]. Demographic data were obtained by standardized interviewer assisted questionnaires.

### Genetic analysis

Genomic DNA was extracted from peripheral blood samples using standard procedures. A total of ten SNPs in the *ARMS2*, *CFH*, *LIPC*, *CETP*, *APOE*, *FADS1*, *LPL*, and *ABCA1* genes (see **S1 Table**) were genotyped using the KASPar SNP Genotyping System by LGC Genomics.

### Lipid mass spectrometry

For lipid analysis, cells were harvested in 0.1% sodium dodecyl sulfate (SDS) and protein content quantified using BCA protein assay (Pierce). Lipids from cell extracts and serum samples were extracted according to the method by Bligh and Dyer [45] in the presence of non-naturally occurring lipid species used as internal standards (PC 14:0/14:0, PC 22:0/22:0, PE 14:0/14:0, PE 20:0/20:0 (di-phytanoyl), LPC 13:0, LPC 19:0, Cer d18:1/14:0, Cer d18:1/17:0, D7-free cholesterol (FC), CE 17:0 and CE 22:0).

Sphingolipid species were quantified by ESI-MS/MS using methods validated and described previously [31]. Serum samples were processed in two different batches. In brief, samples were analyzed by direct flow injection on a Quattro Ultima triple quadrupole mass spectrometer (Micromass, Manchester, UK) by direct-flow injection analysis using a HTS PAL autosampler (Zwingen, Switzerland) and an Agilent 1100 binary pump (Waldbronn, Germany) with a solvent mixture of methanol containing 10 mM ammonium acetate and chloroform (3:1, v/v). A flow gradient was performed starting with a flow of 55 mL/min for 6 s followed by 30 mL/min for 1.0 min and an increase to 250 mL/min for another 12 s.

A precursor ion scan of *m/z* 184 specific for phosphocholine containing lipids was used for PC, SM, and LPC. Ceramides were analyzed similarly to a previously described methodology [46] using N-heptadecanoyl-sphingosine as internal standard.

Quantification was achieved by calibration lines generated by addition of naturally occurring lipid species to serum. All lipid classes were quantified with internal standards belonging to the same lipid class, except SM (PC internal standards). Calibration lines for sphingolipids were generated for the following naturally occurring species: SM 34:2, 36:2, 36:1; Cer d18:1/16:0, 18:0, 20:0, 24:1, 24:0. These calibration lines were also applied for not calibrated species, as follows: concentrations of saturated, monounsaturated, and polyunsaturated species were calculated using the closest related saturated, monounsaturated, and polyunsaturated calibration line slope, respectively.

Correction of isotopic overlap of lipid species as well as data analysis was performed by self-programmed Excel macros for all lipid classes according to the principles described previously [47]. Lipid species were annotated according to the “Shorthand Notation for Lipid Structures Derived from Mass Spectrometry” [48]. SM species annotation is based on the assumption that the main base contains two hydroxyl groups.

### Malondialdehyde-acetaldehyde (MAA)-BSA adducts

Albumin bovine Fraction V (BSA fatty acid free, SERVA) was incubated with 20 mM malondialdehyde tetrabutylammonium salt (MDA; Sigma-Aldrich, St. Louis, MO, USA) and 20 mM acetaldehyde water free (Sigma-Aldrich) in 50 mM sodium phosphate buffer (pH 7.2) at 37°C for 24 h to produce MAA-BSA. Unreacted aldehydes were removed by extensive dialysis against PBS. BSA used as control of MAA-BSA in the experiments was treated in parallel with the same buffers and under the same conditions than MAA-BSA but excluding the addition of aldehydes. Increase in mobility due to loss of positive charge in reaction with malondialdehyde was observed in native PAGE. The degree of modification was assessed by the amount of specific MAA fluorescence present (Ex. 430/10 nm, Em. 480/10 nm). MAA specific modifications were corroborated by indirect enzyme-linked immunosorbent assay (ELISA) using the 1F83 monoclonal antibody against MDHDC (4-methyl-1,4-dihydropyridine-3,5-dicarbaldehyde) [49]. BSA treated with 1.33 or 20 mM malondialdehyde (MDA-BSA), and commercial MDA-BSA (Academy Bio-Medical Company, Houston, USA) were used for comparison purposes (**S1 Fig**).

### BSA acetylation

BSA was acetylated with acetic anhydride according to a method by Basu et al. [50] with some modifications. Briefly, 0.5 mL of 0.15 M NaCl solution of BSA (15 mg/mL) was added to 0.5 mL of sodium-acetate saturated solution. Then multiple small aliquots of anhydride acetic were added over a period of 1 h until reaching a proportion of 2 μL of acetic anhydride per mg of BSA. The mixture was incubated an additional period of 2 h, and then dialyzed against 0.15 M NaCl for 24 h and further in PBS for another 24 h. BSA used as control of acetylated BSA (acetyl-BSA) was treated under the same conditions but without adding acetic anhydride. The higher mobility of acetyl-BSA in comparison to BSA in native PAGE indicates the loss of positive charge (**S2 Fig**).

### Complement proteins

Expression of recombinant FHL-1 Y402 and H402 in *Pichia pastoris* or baculovirus expression system was previously described [51,52]. Purified human CFH from two different batches was purchased from CompTech (Tyler, USA) and, according to the company, 16 or more serum units were used as starting material for the protein isolation.

### Cell culture and treatments

WERI-Rb1 cells (HTB-169, obtained from ATCC Manassas, VA, USA) were cultured in RPMI-1640 medium supplemented with 10% fetal bovine serum (FBS) and 100 U/mL penicillin/streptomycin (P/S). All media and cell culture supplies were purchased from Life Technologies (Carlsbad, CA, USA). Cells were grown in an incubator at 37°C in 5% CO_2_. For experiments, WERI-Rb1 cells (4×10^5^ cells/cm^2^) were plated on wells coated with poly-L-lysine (0.1 mg/mL, Sigma-Aldrich) and cultured for 48 h before starting the experiments. Then, cells were serum starved for 24 h and treated with MAA-BSA, acetyl-BSA or BSA for another 24 h in serum-free medium. For CFH and FHL-1 treatments, medium containing 80 μg/mL BSA or MAA-BSA and 200 μg/mL CFH, 80 μg/mL FHL-1 or PBS was pre-incubated for 30 min at RT and added to the cells for 24 h.

### MTT assay

Cells were incubated in media with 0.5 mg/mL Thiazolyl Blue Tetrazolium Bromide (MTT, Sigma-Aldrich) for 30 min at 37°C. Cell media was carefully removed and 4 mM HCl in isopropanol added. After 15 min of incubation under darkness at room temperature, absorbance of the supernatant at 540 nm was measured in a Varioskan Flash Reader (Thermo Scientific).

### RNA isolation and PCR

Total RNA was isolated from cultured cells using the RNeasy Mini Kit (Qiagen, Hilden, Germany) according to the manufacturer’s recommendations. Genomic DNA was removed by DNAse treatment (Roche, Mannheim, Germany). The RNA was quantified using a ND-1000 NanoDrop Spectrophotometer (PeqLab, Erlangen, Germany) and stored at -80°C. First-strand cDNAs from 1 μg of total RNA were synthesized using the RevertAid H Minus First-Strand cDNA Synthesis Kit (Fermentas, St. Leon-Rot, Germany) and random hexamer oligonucleotide primers. For RT-PCR, 50 ng of cDNA was used as templates for PCR with Go Taq Polymerase (Promega, Mannheim, Germany) at a final volume of 25 μL. PCR products were electrophoretically separated in a 2% agarose gel. For quantitative RT-PCR, amplification of 50 ng cDNA was performed with an ABI7900HT machine (Applied Biosystems, Darmstadt, Germany) in 10 μL reactions containing 1x TaqMan Universal PCR Master Mix (Applied Biosystems, Darmstadt, Germany), 200 nM of primers and 0.25 μL of dual-labeled probe (Roche ProbeLibrary, Roche Applied Science, Mannheim, Germany). Measurements were performed in triplicates and results were analyzed with an ABI sequence detector software version 2.3 (Applied Biosystems, Darmstadt, Germany) applying the ΔΔCt method for relative quantification. Rates of mRNA expression were normalized to *HPRT1*. For each qPCR run and gene, the lowest value was set to 1 and all the other values scaled accordingly. Primer sequences for amplification of target genes are listed in **S2 Table**.

### Data analysis

From the two lipid classes measured, 17 SM and 9 ceramides fulfilled the retention criteria of: a) <5% of cases with a value of zero, and b) lipid species representing >0.5% of the lipid class. For each lipid species, we carried out a linear regression analysis corrected for age, sex and batch between controls and late AMD patients. Within each lipid class, we corrected for multiple comparisons using the Benjamini-Hochberg (BH) method [53]. A subsequent linear regression analysis that consider the two different late AMD stages was performed to evaluate differences between controls and GA or CNV patients. Logistic regression was used to calculate odd ratios controlling for age, sex and batch. For sensitivity analysis, logistic regressions adjusted for age, sex, batch and genetic polymorphisms were performed in a reduced dataset of genotyped subjects (Control, n = 94; GA, n = 31; CNV, n = 161). Regression analyses were carried out using R software (r-project.org).

Pearson’s χ^2^ test for categorical data, analyses of variance (ANOVAs), and two-tailed Student’s *t*-tests were done using the Infostat 2011 Software (UNC, Córdoba, Argentina). Blocked ANOVAs and paired *t*-tests were carried out when necessary. Normality and homogeneity of variance were tested using Shapiro-Wilks and Levene tests, respectively.

## Results

### Sphingolipid species in AMD serum and the influence of genetic variants

Levels of ceramides and SM were measured in serum samples from 244 late stage AMD patients and 129 age-matched healthy controls (**Table 1**). Multiple linear regression analyses adjusting for age, sex and batch effect were performed, with corrections for multiple comparisons within each of the two lipid classes (**Table 2, S3 Table**). In the joint late stage AMD analysis, we found higher levels of hexosylceramide (HexCer) d18:1/16:0 in AMD patients compared to controls (**Table 2**). Hexosylceramide species include both glucosylceramide and galactosylceramide species, which are ceramides with a glucose or galactose residue, respectively. When separating the two late stage forms of AMD, we found higher levels of Cer d18:1/16:0 specifically in GA patients compared to controls (**Table 2**).

**Table 1:** Characteristics of the study subjects.

	Controls	Geographic atrophy	Choroidal neovascularization
**n**	129	47	197
**Mean Age ± SD, y**	77.2 ± 8.4	78.9 ± 9.8	77.8 ± 8.9
**Age-range**	59–97	57–95	56–98
**Female sex, %**	55.8	57.4	62.9

No significant differences among the groups were observed for age (AMD vs. control: Student’s *t*-test, T = -0.86, p = 0.39; GA vs. CNV vs. control: ANOVA, F = 0.62, p = 0.54) or sex (Pearson’s χ^2^; AMD vs. control: χ^2^ = 1.29, p = 0.26; GA vs. CNV vs. control: χ^2^ = 1.77, p = 0.41).

**Table 2:** Measurement of sphingolipid species in serum of late stage AMD patients with GA or CNV compared to healthy controls.

	Controls	All late stage AMD		GA		CNV	
Lipid species	Mean ± SD (μM)	Mean ± SD (μM)	p^a^	Mean ± SD (μM)	p^a^	Mean ± SD (μM)	p^a^
**Ceramides**							
Cer d18:1/16:0	0.69 ± 0.24	0.74 ± 0.25	**0.0249**	0.83 ± 0.31	**0.0041**^**b**^	0.72 ± 0.24	0.1307
Cer d18:1/18:0	0.27 ± 0.08	0.29 ± 0.08	0.0749	0.28 ± 0.07	0.5131	0.30 ± 0.08	0.0578
Cer d18:1/20:0	0.29 ± 0.14	0.31 ± 0.13	0.2266	0.34 ± 0.18	**0.0276**	0.30 ± 0.12	0.5971
Cer d18:1/22:0	1.22 ± 0.38	1.35 ± 0.44	0.1752	1.20 ± 0.35	0.5058	1.38 ± 0.45	0.1704
Cer d18:1/23:0	1.18 ± 0.40	1.26 ± 0.41	0.8171	1.11 ± 0.29	0.8037	1.30 ± 0.43	0.6957
Cer d18:1/24:1	1.74 ± 0.59	1.87 ± 0.57	0.4423	1.78 ± 0.50	0.2760	1.90 ± 0.58	0.6397
Cer d18:1/24:0	3.27 ± 1.11	3.66 ± 1.33	0.4152	2.97 ± 0.85	0.7694	3.83 ± 1.38	0.2603
HexCer d18:1/16:0	0.53 ± 0.17	0.64 ± 0.22	**0.0009**^**b**^	0.57 ± 0.18	**0.0478**	0.66 ± 0.22	**0.0013**^**b**^
HexCer d18:1/24:1	0.67 ± 0.23	0.76 ± 0.27	0.0766	0.67 ± 0.27	0.4043	0.78 ± 0.27	0.0708
**Sphingomyelins**							
SM 32:1	12.56 ± 4.45	12.71 ± 4.17	0.3365	13.78 ± 3.70	0.2418	12.45 ± 4.24	0.5035
SM 33:1	6.88 ± 2.48	7.17 ± 2.53	0.1214	7.69 ± 2.00	0.1535	7.04 ± 2.63	0.2013
SM 34:2	23.55 ± 6.71	24.36 ± 6.79	0.1919	26.13 ± 5.65	**0.0406**	23.94 ± 6.98	0.4760
SM 34:1	111.60 ± 30.59	116.55 ± 29.99	**0.0379**	122.97 ± 22.87	0.0711	115.02 ± 31.30	0.0783
SM 34:0	5.64 ± 2.07	6.24 ± 2.12	0.3267	5.73 ± 1.79	0.2830	6.37 ± 2.18	0.4623
SM 35:1	5.70 ± 2.07	5.97 ± 2.21	0.0871	6.41 ± 1.73	0.1305	5.87 ± 2.30	0.1500
SM 36:2	15.37 ± 5.42	15.96 ± 5.07	0.2026	16.77 ± 4.29	0.1571	15.77 ± 5.23	0.3437
SM 36:1	22.38 ± 7.00	23.59 ± 7.32	**0.0261**	24.64 ± 5.66	0.1319	23.34 ± 7.66	**0.0384**
SM 38:2	8.18 ± 2.86	8.47 ± 2.86	0.1600	8.87 ± 2.87	0.2454	8.37 ± 2.86	0.2239
SM 38:1	14.34 ± 6.19	16.58 ± 6.62	0.1822	13.37 ± 5.03	0.8302	17.34 ± 6.73	0.1234
SM 40:2	32.97 ± 9.54	33.69 ± 9.17	0.5561	34.80 ± 7.53	0.2340	33.42 ± 9.51	0.8379
SM 40:1	25.76 ± 7.82	27.52 ± 7.62	0.1619	26.44 ± 6.24	0.2954	27.78 ± 7.91	0.2082
SM 41:2	16.18 ± 5.52	16.48 ± 4.97	0.1853	18.11 ± 3.93	0.0620	16.09 ± 5.12	0.4153
SM 41:1	12.35 ± 3.91	12.48 ± 3.62	0.2863	13.17 ± 3.10	0.2953	12.31 ± 3.73	0.3946
SM 42:3	33.42 ± 10.05	34.68 ± 10.06	0.1663	38.65 ± 9.87	**0.0035**	33.73 ± 9.90	0.6625
SM 42:2	78.50 ± 21.30	81.13 ± 23.23	**0.0421**	89.94 ± 22.58	**0.0096**	79.03 ± 22.95	0.1698
SM 42:1	16.80 ± 4.78	17.68 ± 4.82	0.0814	17.93 ± 4.13	0.1230	17.61 ± 4.98	0.1428

^a^p-value of the linear regression analysis adjusted for age, sex and batch effect (versus controls). p-values < 0.05 are in bold font.

^b^p-values that remained significant after adjusting for multiple comparisons within each lipid class using the Benjamini-Hochberg method.

In addition, we carried out logistic regression analyses to evaluate the odds of AMD per unit increase in ceramide species, adjusting for age, sex and batch effect. Increasing Cer d18:1/16:0 (OR = 4.89, 95% CI 1.37–18.32, p = 0.016) and HexCer d18:1/16:0 (OR = 11.00, 95% CI 2.80–47.15, p = 0.00086) were significantly associated with increasing odds of suffering from GA or late AMD (i.e. GA + CNV), respectively.

**Fig 1:**
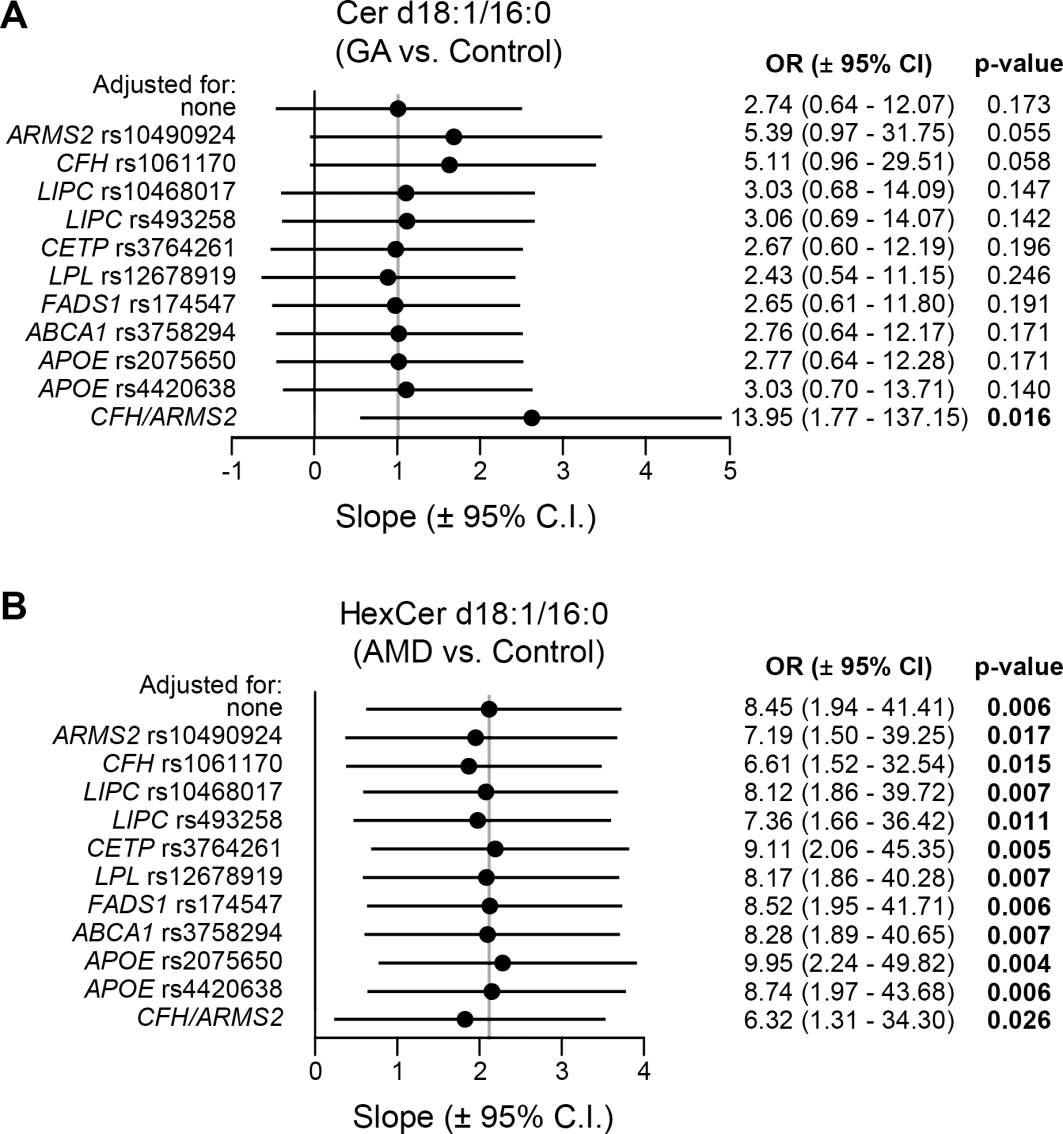
Sensitivity analysis of ceramide species against selected AMD associated genetic variants. A subgroup of genotyped samples including controls (n = 94), geographic atrophy (GA, n = 31) and choroidal neovascularization (CNV, n = 161) was used to determine the influence of genetic variants in the association of lipid species and disease by multiple logistic regression. Regressions were adjusted for age, sex, batch and genetic variants for: **(A)** Ceramide (Cer) d18:1/16:0 association with GA, **(B)** Hexosylceramide (HexCer) d18:1/16:0 association with all late AMD (i.e. AMD = GA + CNV). The vertical grey lines indicate the slope of the regression non-adjusted for genetic variants. Odd ratios (OR) ± 95% confidence intervals (CI) are shown together with the p-values of the regressions. *CFH*/*ARMS2*: adjusted for both *CFH* rs1061170 and *ARMS2* rs10490924.

We next carried out a sensitivity analysis to evaluate the influence of AMD genetic risk variants in the association between serum ceramide species and disease state. We used a reduced dataset of genotyped subjects (Control, n = 94; GA, n = 31; CNV, n = 161), and included genetic variants with strong effects on AMD risk (rs10490924:*ARMS2* and rs1061170:*CFH*) [54], in addition to variants in genes from lipid metabolism (**S1 Table**). Genetic variants in lipid-related genes appear not to influence the associations observed (**Fig 1**). In contrast, variants rs10490924 and rs1061170 tend to increase the association between Cer d18:1/16:0 and GA (**Fig 1A**). Moreover, a model adjusted for these two variants showed a significant difference between GA and controls in the reduced dataset (**Fig 1A**).

### MAA adducts regulate the ceramide metabolism in WERI-Rb1

**Fig 2:**
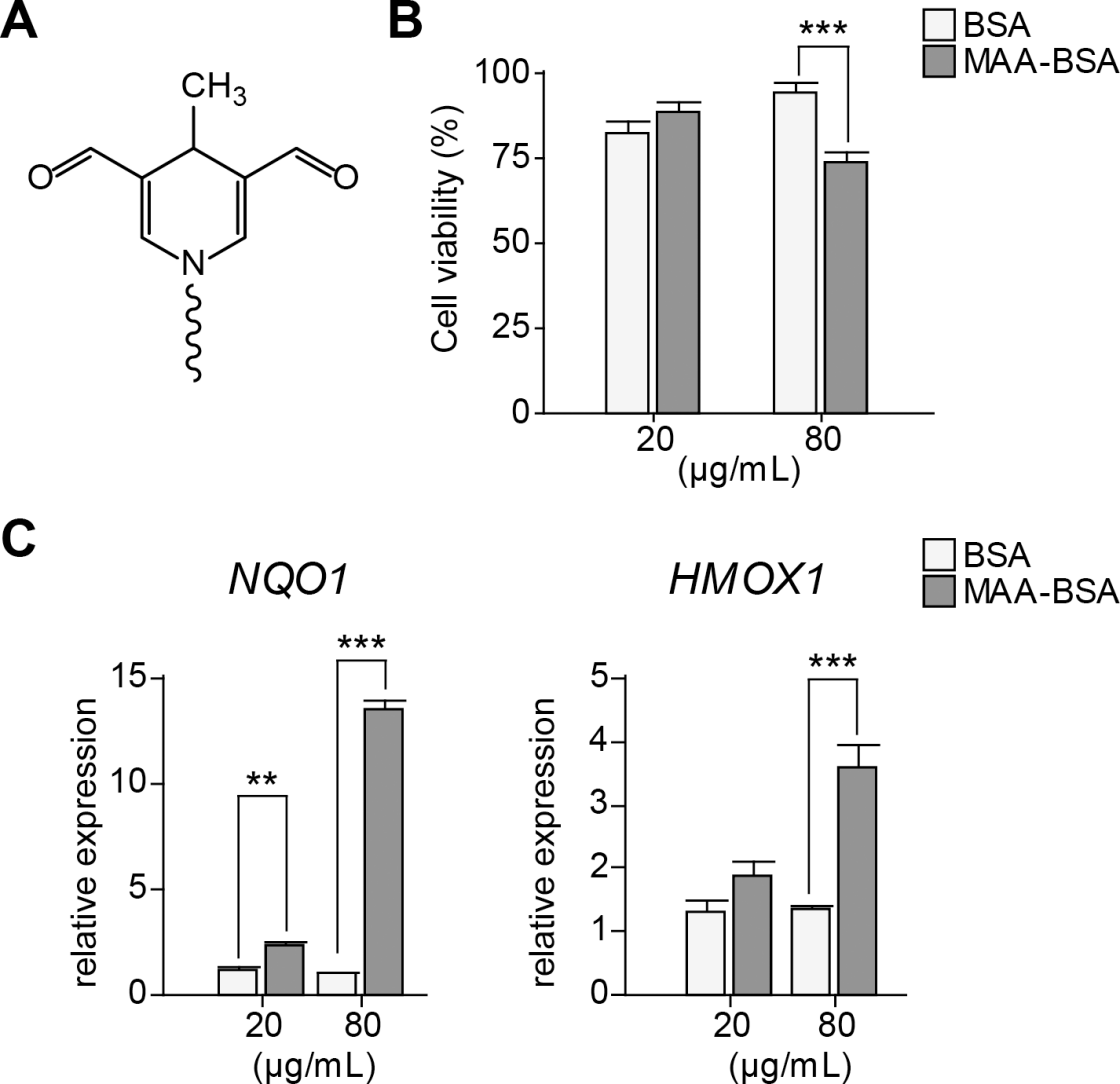
Cell survival and alteration of the stress response under MAA-BSA treatment in WERI-Rb1 cells. **(A)** Schematic structure of MDHDC (4-methyl-1,4-dihydropyridine-3,5-dicarbaldehyde), the most prominent adduct in malondialdehyde-acetaldehyde (MAA) protein modifications. **(B-C)** Cells treated with 20 or 80 μg/mL of BSA or MAA-BSA for 24 h. **(B)** Cell viability (%) measured by MTT assay; the repetition with the highest value was considered as 100% (each value corresponds to the mean ± SEM of six-fold independently performed replicates). **(C)** Gene expression of NAD(P)H dehydrogenase [quinone] 1 (*NQO1*) and Heme oxygenase 1 (*HMOX1*) (each value corresponds to the mean ± SEM of three-fold independently performed replicates). Statistics: 2-way ANOVA and simple effect test (when significant interaction). ***p<0.001, **p<0.01.

Ceramide metabolism in photoreceptor cells has previously been shown to be altered under oxidative-stress conditions [55,56]. To establish a cell-based assay for testing the modulation of sphingolipid gene expression, we investigated the possibility that MAA protein adducts regulate the synthesis of these lipids in the human cell line WERI-Rb1, an early stage cone lineage cell line [57]. First, WERI-Rb1 cells were treated with 20 and 80 μg/mL of MAA-BSA adducts (**Fig 2A**) to check for the influence of these oxidation-derived adducts in cell survival and activation of the cellular stress response. Cells exposed to 80 μg/mL MAA-BSA showed a reduced cell survival compared to BSA control (**Fig 2B**), and induction of the stress response as indicated by an increase in gene expression of NAD(P)H dehydrogenase [quinone] 1 (*NQO1*) and Heme oxygenase 1 (*HMOX1*) (**Fig 2C**).

**Fig 3:**
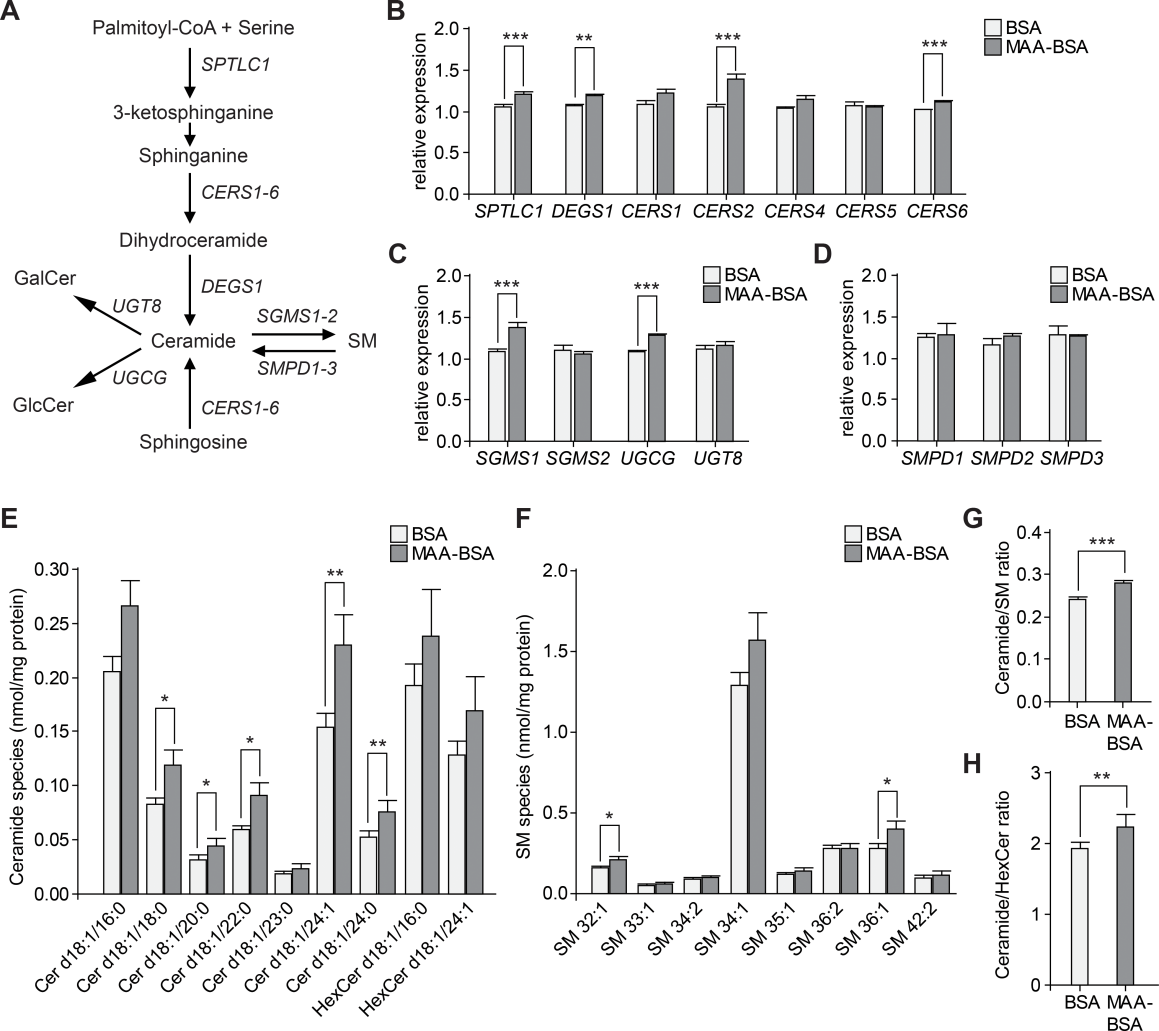
Alteration of the sphingolipid metabolism under MAA-BSA treatment in WERI-Rb1 cells. **(A)** The sphingolipid pathway depicting the gene symbols of the enzymes involved (complete protein names in **S2 Table**). **(B-H)** Cells were treated with 80 μg/mL of BSA or MAA-BSA for 24 h, and gene expression or lipid levels were measured. **(B)** Genes from the *de novo* and salvage ceramide synthesis pathways. **(C)** Genes involved in the synthesis of SM from ceramides, and synthesis of glucosylceramides (GlcCer) and galactosylceramides (GalCer), both hexosylceramides (HexCer). **(D)** Genes involved in the generation of ceramide by sphingomyelin (SM) hydrolysis. **(E)** Ceramide (Cer) species levels. **(F)** SM species levels; only results from most abundant species are shown. **(G)** Ratio of total ceramide to total SM levels. **(H)** Ratio of total ceramide to total HexCer levels. Each value corresponds to the mean ± SEM of six-fold independently performed replicates evaluated in two batches. Statistics: Linear regression adjusted for batch of analysis; ***p<0.001, **p<0.01, *p<0.05.

We then evaluated gene expression of selected enzymes involved in ceramide metabolism (**Fig 3A** and **S3 Fig**). From *de novo* and salvage ceramide synthesis pathways, expression of *SPTLC1*, *DEGS1*, *CERS2* and *CERS6* was upregulated under MAA-BSA treatment compared to BSA control (**Fig 3B**). Additionally, gene expression of *SGMS1* involved in SM synthesis, and *UGCG* involved in glucosylceramide synthesis, one of the two types of hexosylceramides, were also upregulated under MAA-BSA treatment (**Fig 3C**). No significant differences were observed for the gene expression of sphingomyelinases (*SMPD1-3*) that synthesize ceramide by hydrolysis of SM (**Fig 3D**). Mass spectrometry measurements confirmed the increase in ceramide species, and also a tendency for increased hexosylceramide species under MAA-BSA treatment (HexCer d18:1/16:0, p = 0.18; HexCer d18:1/24:1, p = 0.11) (**Fig 3E**). Levels of two SM species were also significantly increased under MAA treatment (**Fig 3F**). MAA-BSA produced a significant increase in *UGCG* and *SGMS1* gene expression levels as a possible response to reduce ceramide toxicity by generating less toxic sphingolipid species. However, this was not enough to counteract the increase in ceramide levels, as the ratios of total ceramide to total SM or total hexosylceramide were greater in MAA-BSA than in BSA treated cells (**Fig 3G and 3H**).

To rule out the possibility that the regulation of genes by MAA-BSA may be associated with the loss of positive charge as a consequence of MAA adduction on amino groups, we treated WERI-Rb1 cells with 80 μg/mL of acetylated-BSA (acetyl-BSA) or BSA. Acetylation of proteins was shown to block amino groups and remove positive charges from proteins [50,58]. We observed no significant differences between acetyl-BSA and BSA treated cells in the expression of *NQO1* or genes in the ceramide synthesis pathway that were consistently increased by MAA-BSA in comparison to BSA treatments (S2 Fig). Therefore, the loss of positive charge in BSA is not sufficient to explain the effects of MAA-BSA on WERI-Rb1 gene expression.

### Risk and non-risk isoforms of FHL-1 differentially regulate gene expression of ceramide metabolism

**Fig 4:**
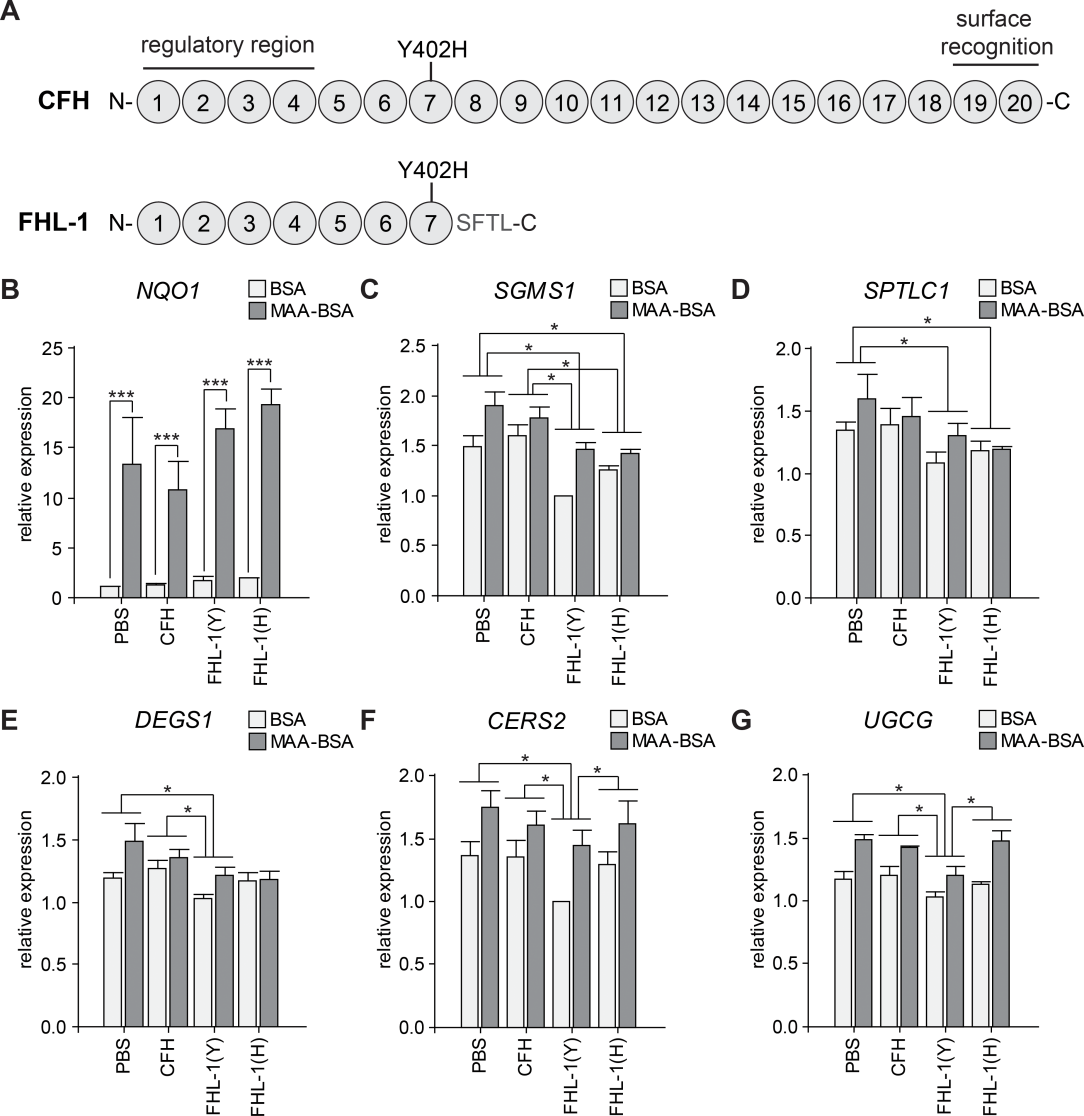
Influence of FHL-1 variants in expression of ceramide metabolism genes. **(A)** Schematic representation of CFH and FHL-1 proteins. CFH is composed of 20 short consensus repeats (SCR), and its alternative splicing variant FHL-1 of 7 SCR and a unique extension of 4 amino acids. The complement regulatory region (SCR 1–4), and surface recognition domains (SCR 19–20) are indicated. Residue 402 is located in the SCR 7 of both proteins. **(B-G)** WERI-Rb1 cells treated with 80 μg/mL BSA or MAA-BSA during 24 h with or without addition of 200 μg/mL CFH or 80 μg/mL FHL-1 proteins. Expression of genes from **(B)** NAD(P)H dehydrogenase [quinone] 1 (*NQO1*), **(C)** Phosphatidylcholine:ceramide cholinephosphotransferase 1 (*SGMS1*), **(D)** Serine palmitoyltransferase 1 (*SPTLC1*), **(E)** Sphingolipid delta(4)-desaturase DES1 (*DEGS1*), **(F)** Ceramide synthase 2 (*CERS2*), **(G)** Ceramide glucosyltransferase (*UGCG*). Each value corresponds to the mean ± SEM of four-fold independently performed experiments. Statistics: two-way blocked ANOVA and Tukey’s test (no significant interaction between principal effects was observed); (C-G) only the significance from the Tukey’s test comparing PBS, CFH, FHL-1:Y402 (Y), and FHL-1:H402 (H) are shown in the graphs; all genes showed significant differences between MAA-BSA and BSA treatments: (C, F) p<0.001, (G, E) p<0.01, (D) p<0.05. ***p<0.001, *p<0.05.

To test whether variant rs1061170:*CFH* (p.Y402H) differentially influences the regulation of ceramide metabolism in WERI-Rb1 cells, we used two variants of recombinant FHL-1, a splicing variant of CFH shown to be the predominant complement regulator in Bruch's membrane [59] (**Fig 4A**). Full-length CFH isolated from pooled human serum was included as a control of FHL-1 effects on cells. MAA-BSA or BSA were pre-incubated with FHL-1:Y402, FHL-1:H402 or CFH in an equimolar concentration, and then added to WERI-Rb1 cells for 24 h. Quantitative RT-PCR analysis revealed that *NQO1* gene expression was higher in MAA-BSA compared to BSA in spite of FHL-1 or CFH addition, showing that the stress response under MAA-BSA was not affected by these complement proteins (**Fig 4B**). Gene expression of *SGMS1* and *SPTLC1*, the rate limiting enzyme of *de novo* ceramide synthesis, was significantly decreased under the influence of both FHL-1 variants compared to the PBS control (**Fig 4C and 4D**). *DEGS1*, which is also participating in *de novo* ceramide synthesis, showed a significant decrease of expression for non-risk associated variant FHL-1:Y402 compared to PBS and CFH (**Fig 4E**). Finally, our results demonstrated that FHL-1:Y402, but not the AMD risk variant FHL-1:H402 or the full-length CFH, significantly downregulated the expression of *CERS2*, which participates in *de novo* and salvage ceramide synthesis pathways, and of *UGCG*, involved in glucosylceramide synthesis, in the presence and absence of MAA-BSA (**Fig 4F and 4G**).

## Discussion

Here, we show for the first time that levels of ceramide species are significantly elevated in serum of late stage AMD patients compared to healthy control individuals in a population based study. We observed a 1.20-fold increase of Cer d18:1/16:0 in GA and a 1.21-fold increase of HexCer d18:1/16:0 in all late AMD compared to controls. The differences reported here for AMD are in accordance to fold-changes in ceramides observed in other neurodegenerative disorders. For example, Han et al. [41] showed that plasma Cer d18:1/16:0 was approximately 1.27-fold significantly increased in AD patients compared to controls, while a more recent study that evaluated plasma of autopsy-confirmed AD dementia patients reported a 1.21-fold increase of the same ceramide species [60]. Further studies in different cohorts of AD patients corroborated the association of circulating ceramides in AD and its neuropsychiatric symptoms [61–63]. Moreover, circulating Cer d18:1/16:0 has been consistently associated with neurological disorders, including Parkinson’s disease, AD, dementia with Lewy bodies and memory impairment [38–41,60–62]. The increased levels of circulating ceramides in AD are consistent with increased levels of these bioactive lipids in cerebrospinal fluid soluble fractions [64] and brains [65] of patients with the disease. It would be of great interest to measure sphingolipid levels in vitreous humor of AMD patients, as the proximity to the retina may give a better understanding of the possible role of these lipids in AMD.

The significant result for higher Cer d18:1/16:0 in GA but not CNV patients compared to controls points to a possible differential role of specific lipid species between AMD late stages. The levels of these ceramide species in plasma seem to depend on the rate of *de novo* synthesis in solid tissues, mainly the liver and adipose tissue [66,67]. Although no correlation analysis was undertaken between ceramide species in plasma and the retina, ratios of omega-3 to omega-6 polyunsaturated fatty acids (PUFA) in serum and phosphatidylethanolamine species containing PUFA in red blood cells were found to be good biomarkers of retinal PUFA levels, including docosahexaenoic acid [68,69]. The lipid composition of plasma has previously been considered as a reflection of the overall lipid metabolism of individuals, although concentrations of lipids between plasma and tissue may differ depending on other factors such as genetics or oxidative-stress generated as a consequence of alcohol consumption and smoking [69].

Our sensitivity analysis revealed no influence of genetic variants in lipid-related genes in the association between ceramides and AMD. However, a suggestive tendency for the influence of AMD-associated variants rs10490924:*ARMS2* and rs1061170:*CFH* was observed for the association between Cer d18:1/16:0 and GA. To better understand such a possible connection, we used an early stage cone lineage cell line, WERI-Rb1, as a model to evaluate sphingolipid modulation at the gene expression level. Previous studies showed an increase in ceramide synthesis in rat retina neuronal cultures exposed to the oxidative stress inducer paraquat [55], and ceramide and/or sphingosine, which is synthetized from ceramide by ceramidases, then trigger apoptosis [70]. In the mouse retina-derived 661W cell line, ceramide was shown to be responsible for the activation of the mitochondrial apoptotic pathway after sodium nitroprusside treatment [56]. Photoreceptors present a high metabolic activity and demand for oxygen and nutrients, and together with the high-energy light that reaches the retina and their high content of PUFA particularly vulnerable to oxidation, they are a main source of peroxides and organic radicals [71]. Malondialdehyde is a by-product of lipid peroxidation previously shown to be present in AMD lesions [72], and circulating levels of malondialdehyde were found elevated in AMD patients compared to controls [73,74]. Therefore, we investigated the possibility that MAA protein adducts may regulate the synthesis of these lipids in WERI-Rb1 cells, to later estimate the effects of FHL-1 variants Y402 and H402 on the gene regulation of the ceramide metabolism.

Here, we demonstrated that treatment of WERI-Rb1 cells with MAA-BSA adducts increased the expression of *SPTLC1*, *DEGS1*, *CERS2*, and *CERS6*, genes from the *de novo* and salvage pathway of ceramide synthesis, and significantly increased the levels of Cer d18:1/18:0, 20:0, 22:0, 24:0, and 24:1. We also observed an increase in *SGMS1* and *UGCG* gene expression suggesting a possible response to hamper ceramide cytotoxicity by further synthesis of the less toxic sphingolipids SM and glucosylceramide from ceramide. Although an increase in glucosylceramide provides an escape route from apoptosis by decreasing ceramide levels, pathological accumulation of glucosylceramide in Gaucher disease results in retinal neuronal cell death [75], and also seems to contribute to the pathogenesis of diabetic retinopathy. In this regard, inhibition of glucosylceramide synthesis in retinal neurons of a diabetic rat model was shown to increase insulin sensitivity and reduce neuronal death [76]. Future investigation of sphingolipids in an AMD animal model may contribute to better understand their role in the disease.

Finally, we focused on the possible regulation of ceramide genes by FHL-1 variant Y402 and AMD risk-associated isoform FHL-1:H402 in WERI-Rb1 cells. We showed that FHL-1:Y402, but not FHL-1:H402 or full-length CFH, decreased the expression of *CERS2* and *UGCG* in the presence and absence of MAA-BSA. CFH variant H402 was previously shown to have an impaired ability to bind MAA-BSA compared to CFH variant Y402 [72], and therefore we expected FHL-1:Y402 incubated with MAA-BSA adducts to reduce the effect of this peroxidation product when compared to FHL-1:H402. However, the binding of FHL-1 to MAA-BSA adducts in WERI-Rb1 cell media is not enough to explain the differential influence of FHL-1 variants in *CERS2* and *UGCG* gene expression, as the effect was observed also for the BSA control treatment. A similar decrease of relative expression under FHL-1 Y402 in the BSA control was observed for *SGMS1*, *SPTLC1* and *DEGS1*, although the difference between FHL-1 variants in the expression of these genes was not statistically significant. Moreover, the fact that full-length CFH isolated from pooled serum did not exert the same effect as FHL-1 suggest a specific role of FHL-1 in the modulation of WERI-Rb1 sphingolipid metabolism.

It has been previously shown that FHL-1, but not CFH, promotes cell attachment of mink epithelial-like cell-line CCL64, human melanoma C32 cells and human fibroblast-like MRC-5 cells, even though both proteins display the RGD motif that is necessary for this function at identical positions in SCR 4 [77]. RGD motif is recognized by members of the integrin family of adhesion proteins [78], and it has been demonstrated that inhibition of endothelial cell anchorage by blockade of RGD-binding integrins increases endogenous ceramide [79]. Therefore, it may be possible that FHL-1 promotes regulation of the ceramide metabolism in WERI-Rb1 cells in a membrane receptor-dependent manner using the RGD motif and triggering an intracellular response, which may explain our observation that both FHL-1 variants statistically decreased the gene expression of *SGMS1* and *SPTLC1* compared to PBS control. Moreover, a recent study has shown that the binding of FHL-1 to C-reactive protein and pentraxin-3 differs from the binding of CFH to these proteins, and that the interaction between FHL-1 and pentraxin-3 is altered by the Y402H polymorphism [80]. These results show that FHL-1 has binding abilities different from CFH that are influenced by the Y402H variant. As ceramide synthesis involves three pathways modulated by different stimuli and cellular conditions [81,82], our results showing that only FHL-1 Y402 decreased the gene expression of *CERS2* and *UGCG* compared to controls may be associated with a specific binding ability of this variant to the cell surface and a subsequent response. Further studies are needed to understand the interaction of FHL-1 variants with WERI-Rb1 cells and their regulation of ceramide metabolism.

A recent study demonstrated that C5aR1 regulates glucosylceramide cellular accumulation in experimental and clinical Gaucher disease, which induces complement-activating IgG autoantibodies that drive C5a generation and C5aR1 activation, feeding a cycle of glucosylceramide accumulation and immune response activation [83]. Another study demonstrated that two isoforms of the ceramide transporter protein (CERT) bind to C1q and initiate the classical complement pathway in normal human serum, and that C1q binds to the longer splicing isoform CERT_L_ in the surface of apoptotic cells [84]. The results from these studies and our findings that FHL-1 regulates the gene expression of ceramide metabolism in WERI-Rb1 cells highlight the importance to further investigate the connection between the sphingolipid metabolism and the complement system. Future experiments should evaluate the regulation of sphingolipids by FHL-1 in the presence of other complement proteins to better understand the possible *in vivo* implication of these findings.

In conclusion, our study reports for the first time higher levels of serum ceramides in AMD patients that may reflect alterations in the sphingolipid metabolism as a consequence of pro-oxidant environments. Moreover, the genetic variant rs1061170 (p.Y402H) in *CFH* which is a strong risk factor for AMD seems to influence the levels of ceramides in serum, and FHL-1 variants Y402 and H402 differentially regulate the expression of ceramide synthesis genes in retinoblastoma-derived cells. These findings point to a possible influence of *CFH* variant rs1061170 in cellular processes regulated by sphingolipid levels, such as cell survival, proliferation and autophagy.

## Supporting information

S1 Table Selected SNPs for the sensitivity analysis with reference papers. (DOC)

S2 Table Sequence of primers used for PCR analysis. (DOC)

S3 Table Linear regression association of serum sphingolipid species to late age-related macular degeneration (AMD) stages. (DOC)

S1 Fig Corroboration of malondialdehyde-acetaldehyde protein adducts synthesis. **(A)** Native PAGE of bovine serum albumin (BSA), BSA treated with 1.33 or 20 mM malondialdehyde (MDA-BSA), and BSA modified with 20 mM MDA and 20 mM acetaldehyde (MAA-BSA) stained with Coomassie Blue. **(B)** Fluorescence intensity of 10 μg of unmodified BSA, MDA-BSA and MAA-BSA proteins (Ex. 430/10 nm, Em. 480/10 nm). **(C)** MAA specific modifications were corroborated by indirect enzyme-linked immunosorbent assay (ELISA). Maxisorp plates were coated with a dilution series of each protein and wells were incubated with 1F83 monoclonal antibody (mAb) against MDHDC (4-methyl-1,4-dihydropyridine-3,5-dicarbaldehyde). 1F83 was detected with HRP-conjugated Ab. MDA-BSA comm.: commercial MDA-BSA. **(D)** Fluorescence intensity of unmodified BSA and MAA-BSA proteins from four synthesis batches (mean ± SEM). **(E)** Binding of MDHDC mAb against unmodified BSA and MAA-BSA proteins from four synthesis batches (mean ± SEM).
(DOC)

S2 Fig Influence of acetylated-BSA (acetyl-BSA) in gene expression of WERI-Rb1 cells. **(A)** Native PAGE of bovine serum albumin (BSA) and BSA treated with 2 μL of acetic anhydride per mg of protein to produce acetyl-BSA. The gel was stained with Coomassie Blue. The table shows the retention factor relative to BSA (Rf_BSA_) calculated as the ratio of the distance each protein has travelled over the distance travelled by BSA. Rf_BSA_ for MAA-BSA was obtained from the gel showed in S1 Fig. **(B-C)** WERI-Rb1 cells treated with 80 μg/mL of BSA or acetyl-BSA for 24 h. **(B)** Gene expression of NAD(P)H dehydrogenase [quinone] 1 (*NQO1*) (each value corresponds to the mean ± SEM of three-fold independently performed experiments). **(C)** Gene expression of serine palmitoyltransferase 1 (*SPTLC1*), sphingolipid delta(4)-desaturase DES1 (*DEGS1*), ceramide synthase 2 (*CERS2*), and ceramide synthase 6 (*CERS6*) (each value corresponds to the mean ± SEM of three-fold independently performed experiments). Statistics: Paired Student’s *t*-test, p>0.05 for all genes evaluated.
(DOC)

S3 Fig Expression of selected genes in the ceramide metabolism in WERI-Rb1 cells. Expression of 15 genes from the ceramide metabolism evaluated by RT-PCR in retinoblastoma cell line WERI-Rb1. *CERS3* presented a very low expression and was not included in later experiments. Ctrl lanes for each gene represent a control without cDNA addition. The lower molecular weight band in *UGCG* and Ctrl *UGCG* lanes correspond to primer dimers.
(DOC)
