# Supplementary material for: Evaluation of serum sphingolipids and the influence of genetic risk factors in age-related macular degeneration
Source: PLoS One. 2018 Aug 2;13(8):e0200739. doi: 10.1371/journal.pone.0200739 (PMC6071970; doi:10.1371/journal.pone.0200739)
Supplement: S1 Table — (DOC) [file pone.0200739.s001.doc]

**S1 Table.** Selected SNPs for the sensitivity analysis with reference papers.

| **Gene** | **SNP** | **References** |
| --- | --- | --- |
| *ARMS2* | rs10490924 |  |
| *CFH* | rs1061170 |  |
| *LIPC* | rs10468017 |  |
| *LIPC* | rs493258 |  |
| *CETP* | rs3764261 |  |
| *LPL* | rs12678919 |  |
| *FADS1* | rs174547 |  |
| *ABCA1* | rs3758294 |  |
| *APOE* | rs2075650 |  |
| *APOE* | rs4420638 |  |

**References**

1. Rivera A, Fisher SA, Fritsche LG, Keilhauer CN, Lichtner P, Meitinger T, et al. Hypothetical LOC387715 is a second major susceptibility gene for age-related macular degeneration, contributing independently of complement factor H to disease risk. Hum Mol Genet. 2005; 14: 3227-3236.

2. Jakobsdottir J, Conley YP, Weeks DE, Mah TS, Ferrell RE, Gorin MB. Susceptibility genes for age-related maculopathy on chromosome 10q26. Am J Hum Genet. 2005; 77: 389-407.

3. Edwards AO, Ritter R, 3rd, Abel KJ, Manning A, Panhuysen C, Farrer LA. Complement factor H polymorphism and age-related macular degeneration. Science. 2005; 308: 421-424.

4. Klein RJ, Zeiss C, Chew EY, Tsai JY, Sackler RS, Haynes C, et al. Complement factor H polymorphism in age-related macular degeneration. Science. 2005; 308: 385-389.

5. Neale BM, Fagerness J, Reynolds R, Sobrin L, Parker M, Raychaudhuri S, et al. Genome-wide association study of advanced age-related macular degeneration identifies a role of the hepatic lipase gene (LIPC). Proc Natl Acad Sci U S A. 2010; 107: 7395-7400.

6. Fritsche LG, Chen W, Schu M, Yaspan BL, Yu Y, Thorleifsson G, et al. Seven new loci associated with age-related macular degeneration. Nat Genet. 2013; 45: 433-439.

7. Chen W, Stambolian D, Edwards AO, Branham KE, Othman M, Jakobsdottir J, et al. Genetic variants near TIMP3 and high-density lipoprotein-associated loci influence susceptibility to age-related macular degeneration. Proc Natl Acad Sci U S A. 2010; 107: 7401-7406.

8. Holliday EG, Smith AV, Cornes BK, Buitendijk GHS, Jensen RA, Sim X, et al. Insights into the Genetic Architecture of Early Stage Age-Related Macular Degeneration: A Genome-Wide Association Study Meta-Analysis. PLoS One. 2013; 8: e53830. 10.1371/journal.pone.0053830.

9. Fritsche LG, Freitag-Wolf S, Bettecken T, Meitinger T, Keilhauer CN, Krawczak M, et al. Age-related macular degeneration and functional promoter and coding variants of the apolipoprotein E gene. Hum Mutat. 2009; 30: 1048-1053.
