# Supplementary material for: Evaluation of serum sphingolipids and the influence of genetic risk factors in age-related macular degeneration
Source: PLoS One. 2018 Aug 2;13(8):e0200739. doi: 10.1371/journal.pone.0200739 (PMC6071970; doi:10.1371/journal.pone.0200739)
Supplement: S2 Table — (DOC) [file pone.0200739.s002.doc]

**S2 Table**. Sequence of primers used for PCR analysis.

| **Gene Symbol** | **Protein name (Uniprot recommended)** | **Forward primer** | **Reverse primer** |
| --- | --- | --- | --- |
| *CERS1* | Ceramide synthase 1 | ctcatcgtctcctcctacgc | actcaagctgcacgtcactg |
| *CERS2* | Ceramide synthase 2 | tgctcttcctcatcgttcg | gcagccgagttttctccttt |
| *CERS3* | Ceramide synthase 3 | ccaggctgaagaaattccag | aacgcaattccagcaacagt |
| *CERS4* | Ceramide synthase 4 | tctctggtgctgctgttacac | tgatactgcatgtagttgaccatc |
| *CERS5* | Ceramide synthase 5 | aaagccttgatcaggggaaa | ttgtgcaggtggtcacatct |
| *CERS6* | Ceramide synthase 6 | cgactgggtatatttcctctctg | ggaagggtaaggtccaacg |
| *DEGS1* | Sphingolipid delta(4)-desaturase DES1 | ggaagacttcgagtgggtctac | ttcatcaaggactttatctctgg |
| *HMOX1* | Heme oxygenase 1 | ggcagagggtgatagaagagg | agctcctgcaactcctcaaa |
| *HPRT1* | Hypoxanthine-guanine phosphoribosyltransferase | tgaccttgatttattttgcatacc | cgagcaagacgttcagtcct |
| *NQO1* | NAD(P)H dehydrogenase [quinone] 1 | gcccagatattgtggctga | accactgcagggggaact |
| *SGMS1* | Phosphatidylcholine:ceramide cholinephosphotransferase 1 | gccaagatgaccagaatgttt | gcgcccaagtattaattcacc |
| *SGMS2* | Phosphatidylcholine:ceramide cholinephosphotransferase 2 | ctacctgtgcctggaatgc | cgttgaacttttgcctgaga |
| *SMPD1* | Sphingomyelin phosphodiesterase | ctatgaagcgatggccaag | tggggaaagagcatagaacc |
| *SMPD2* | Sphingomyelin phosphodiesterase 2 | gagctccccaaccatgaag | gtgcttgctcaagtacggaat |
| *SMPD3* | Sphingomyelin phosphodiesterase 3 | cgtcgtctgtggagatttca | ggtgaacagggagtgttgct |
| *SPTLC1* | Serine palmitoyltransferase 1 | gatgtcagactgcttcaggaaa | cgtgaccacaacccgaat |
| *UGCG* | Ceramide glucosyltransferase | gtttcaatccagaatgatcaggt | cattctgaaattggctcacaaa |
| *UGT8* | 2-hydroxyacylsphingosine 1-beta-galactosyltransferase | agcactggaattcccaagac | tagcaccatttacccatctttg |
