# Supplementary material for: Evaluation of serum sphingolipids and the influence of genetic risk factors in age-related macular degeneration
Source: PLoS One. 2018 Aug 2;13(8):e0200739. doi: 10.1371/journal.pone.0200739 (PMC6071970; doi:10.1371/journal.pone.0200739)
Supplement: S3 Table — (DOC) [file pone.0200739.s003.doc]

**S3 Table.** Linear regression association of serum sphingolipid species to late age-related macular degeneration (AMD) stages.

|  | All late AMD | | | Geographic atrophy | | | Choroidal neovascularization | | |
| --- | --- | --- | --- | --- | --- | --- | --- | --- | --- |
| Lipid species | slope (95% CI)* | p† | pBH^#^ | slope (95% CI)* | p† | pBH^#^ | slope (95% CI)* | p† | pBH^#^ |
| Cer d18:1/16:0 | 0.06 (0.01 - 0.11) | **0.0249** | 0.112 | 0.12 (0.04 - 0.20) | **0.0041** | **0.037** | 0.04 (-0.01 - 0.10) | 0.1307 | 0.294 |
| Cer d18:1/18:0 | 0.02 (0.00 - 0.03) | 0.0749 | 0.172 | 0.01 (-0.02 - 0.04) | 0.5131 | 0.660 | 0.02 (0.00 - 0.04) | 0.0578 | 0.213 |
| Cer d18:1/20:0 | 0.02 (-0.01 - 0.05) | 0.2266 | 0.340 | 0.05 (0.01 - 0.10) | **0.0276** | 0.124 | 0.01 (-0.02 - 0.04) | 0.5971 | 0.696 |
| Cer d18:1/22:0 | 0.06 (-0.03 - 0.14) | 0.1752 | 0.315 | 0.04 (-0.08 - 0.17) | 0.5058 | 0.660 | 0.06 (-0.03 - 0.15) | 0.1704 | 0.307 |
| Cer d18:1/23:0 | 0.01 (-0.07 - 0.09) | 0.8171 | 0.817 | -0.02 (-0.14 - 0.11) | 0.8037 | 0.804 | 0.02 (-0.07 - 0.10) | 0.6957 | 0.696 |
| Cer d18:1/24:1 | 0.05 (-0.07 - 0.16) | 0.4423 | 0.498 | 0.10 (-0.08 - 0.28) | 0.2760 | 0.621 | 0.03 (-0.10 - 0.15) | 0.6397 | 0.696 |
| Cer d18:1/24:0 | 0.09 (-0.13 - 0.30) | 0.4152 | 0.498 | -0.05 (-0.38 - 0.28) | 0.7694 | 0.804 | 0.13 (-0.10 - 0.36) | 0.2603 | 0.390 |
| HexCer d18:1/16:0 | 0.07 (0.03 - 0.11) | **0.0009** | **0.008** | 0.06 (0.00 - 0.12) | **0.0478** | 0.143 | 0.07 (0.03 - 0.11) | **0.0013** | **0.012** |
| HexCer d18:1/24:1 | 0.05 (0.00 - 0.10) | 0.0766 | 0.172 | 0.03 (-0.05 - 0.11) | 0.4043 | 0.660 | 0.05 (0.00 - 0.10) | 0.0708 | 0.213 |
| SM 32:1 | 0.41 (-0.43 - 1.25) | 0.3365 | 0.358 | 0.77 (-0.52 - 2.07) | 0.2418 | 0.314 | 0.30 (-0.59 - 1.20) | 0.5035 | 0.571 |
| SM 33:1 | 0.40 (-0.11 - 0.91) | 0.1214 | 0.265 | 0.57 (-0.21 - 1.35) | 0.1535 | 0.267 | 0.35 (-0.19 - 0.89) | 0.2013 | 0.423 |
| SM 34:2 | 0.91 (-0.46 - 2.27) | 0.1919 | 0.265 | 2.19 (0.09 - 4.29) | **0.0406** | 0.230 | 0.52 (-0.92 - 1.96) | 0.4760 | 0.571 |
| SM 34:1 | 6.54 (0.37 - 12.72) | **0.0379** | 0.238 | 8.76 (-0.76 - 18.29) | 0.0711 | 0.242 | 5.88 (-0.67 - 12.43) | 0.0783 | 0.423 |
| SM 34:0 | 0.21 (-0.21 - 0.62) | 0.3267 | 0.358 | 0.35 (-0.29 - 0.98) | 0.2830 | 0.314 | 0.16 (-0.27 - 0.60) | 0.4623 | 0.571 |
| SM 35:1 | 0.39 (-0.06 - 0.83) | 0.0871 | 0.265 | 0.53 (-0.16 - 1.21) | 0.1305 | 0.267 | 0.34 (-0.13 - 0.81) | 0.1500 | 0.423 |
| SM 36:2 | 0.67 (-0.36 - 1.70) | 0.2026 | 0.265 | 1.15 (-0.44 - 2.74) | 0.1571 | 0.267 | 0.53 (-0.57 - 1.62) | 0.3437 | 0.571 |
| SM 36:1 | 1.64 (0.20 - 3.09) | **0.0261** | 0.238 | 1.72 (-0.52 - 3.95) | 0.1319 | 0.267 | 1.62 (0.09 - 3.16) | **0.0384** | 0.423 |
| SM 38:2 | 0.40 (-0.16 - 0.97) | 0.1600 | 0.265 | 0.51 (-0.36 - 1.38) | 0.2454 | 0.314 | 0.37 (-0.23 - 0.97) | 0.2239 | 0.423 |
| SM 38:1 | 0.76 (-0.36 - 1.88) | 0.1822 | 0.265 | 0.19 (-1.54 - 1.91) | 0.8302 | 0.830 | 0.93 (-0.25 - 2.12) | 0.1234 | 0.423 |
| SM 40:2 | 0.57 (-1.34 - 2.48) | 0.5561 | 0.556 | 1.78 (-1.16 - 4.73) | 0.2340 | 0.314 | 0.21 (-1.81 - 2.24) | 0.8379 | 0.838 |
| SM 40:1 | 1.13 (-0.45 - 2.71) | 0.1619 | 0.265 | 1.30 (-1.14 - 3.74) | 0.2954 | 0.314 | 1.08 (-0.60 - 2.75) | 0.2082 | 0.423 |
| SM 41:2 | 0.69 (-0.33 - 1.70) | 0.1853 | 0.265 | 1.49 (-0.07 - 3.05) | 0.0620 | 0.242 | 0.45 (-0.63 - 1.52) | 0.4153 | 0.571 |
| SM 41:1 | 0.42 (-0.35 - 1.18) | 0.2863 | 0.348 | 0.63 (-0.55 - 1.81) | 0.2953 | 0.314 | 0.35 (-0.46 - 1.16) | 0.3946 | 0.571 |
| SM 42:3 | 1.45 (-0.61 - 3.52) | 0.1663 | 0.265 | 4.71 (1.56 - 7.87) | **0.0035** | 0.059 | 0.48 (-1.69 - 2.65) | 0.6625 | 0.704 |
| SM 42:2 | 4.78 (0.17 - 9.39) | **0.0421** | 0.238 | 9.39 (2.30 - 16.47) | **0.0096** | 0.082 | 3.41 (-1.46 - 8.28) | 0.1698 | 0.423 |
| SM 42:1 | 0.91 (-0.11 - 1.93) | 0.0814 | 0.265 | 1.24 (-0.34 - 2.81) | 0.1230 | 0.267 | 0.81 (-0.27 - 1.89) | 0.1428 | 0.423 |

*Slope (95% confidence interval) and †p-value of the linear regression analysis adjusted for age, sex and batch. p-values < 0.05 are in bold font.

^#^Benjamini-Hochberg adjusted p-value for multiple comparisons within each lipid class. Lipid species with pBH < 0.05 are highlighted in gray.
