# Supplementary material for: Evaluation of serum sphingolipids and the influence of genetic risk factors in age-related macular degeneration
Source: PLoS One. 2018 Aug 2;13(8):e0200739. doi: 10.1371/journal.pone.0200739 (PMC6071970; doi:10.1371/journal.pone.0200739)
Supplement: S1 Fig — (A) Native PAGE of bovine serum albumin (BSA), BSA treated with 1.33 or 20 mM malondialdehyde (MDA-BSA), and BSA modified with 20 mM MDA and 20 mM acetaldehyde (MAA-BSA) stained with Coomassie Blue. (B) Fluorescence intensity of 10 μg of unmodified BSA, MDA-BSA and MAA-BSA proteins (Ex. 430/10 nm, Em. 480/10 nm). (C) MAA specific modifications were corroborated by indirect enzyme-linked immunosorbent assay (ELISA). Maxisorp plates were coated with a dilution series of each protein and wells were incubated with 1F83 monoclonal antibody (mAb) against MDHDC (4-methyl-1,4-dihydropyridine-3,5-dicarbaldehyde). 1F83 was detected with HRP-conjugated Ab. MDA-BSA comm.: commercial MDA-BSA. (D) Fluorescence intensity of unmodified BSA and MAA-BSA proteins from four synthesis batches (mean ± SEM). (E) Binding of MDHDC mAb against unmodified BSA and MAA-BSA proteins from four synthesis batches (mean ± SEM). (DOC) [file pone.0200739.s004.doc]

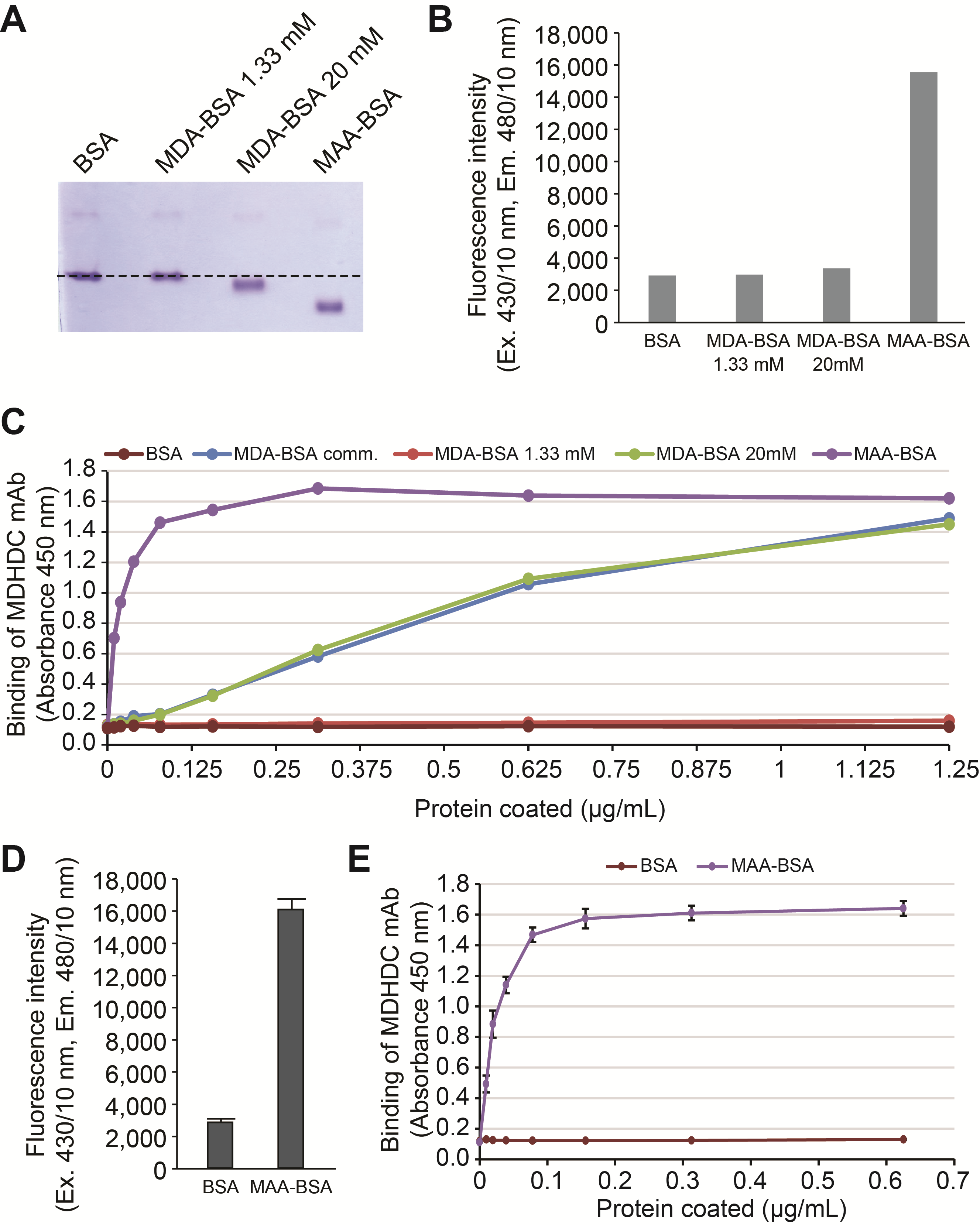


**S1 Fig.** **Corroboration of malondialdehyde-acetaldehyde protein adducts synthesis.** **(A)** Native PAGE of bovine serum albumin (BSA), BSA treated with 1.33 or 20 mM malondialdehyde (MDA-BSA), and BSA modified with 20 mM MDA and 20 mM acetaldehyde (MAA-BSA) stained with Coomassie Blue. **(B)** Fluorescence intensity of 10 μg of unmodified BSA, MDA-BSA and MAA-BSA proteins (Ex. 430/10 nm, Em. 480/10 nm). **(C)** MAA specific modifications were corroborated by indirect enzyme-linked immunosorbent assay (ELISA). Maxisorp plates were coated with a dilution series of each protein and wells were incubated with 1F83 monoclonal antibody (mAb) against MDHDC (4-methyl-1,4-dihydropyridine-3,5-dicarbaldehyde). 1F83 was detected with HRP-conjugated Ab. MDA-BSA comm.: commercial MDA-BSA. **(D)** Fluorescence intensity of unmodified BSA and MAA-BSA proteins from four synthesis batches (mean ± SEM). **(E)** Binding of MDHDC mAb against unmodified BSA and MAA-BSA proteins from four synthesis batches (mean ± SEM).
