# Supplementary material for: Evaluation of serum sphingolipids and the influence of genetic risk factors in age-related macular degeneration
Source: PLoS One. 2018 Aug 2;13(8):e0200739. doi: 10.1371/journal.pone.0200739 (PMC6071970; doi:10.1371/journal.pone.0200739)
Supplement: S2 Fig — (A) Native PAGE of bovine serum albumin (BSA) and BSA treated with 2 μL of acetic anhydride per mg of protein to produce acetyl-BSA. The gel was stained with Coomassie Blue. The table shows the retention factor relative to BSA (RfBSA) calculated as the ratio of the distance each protein has travelled over the distance travelled by BSA. RfBSA for MAA-BSA was obtained from the gel showed in S1 Fig. (B-C) WERI-Rb1 cells treated with 80 μg/mL of BSA or acetyl-BSA for 24 h. (B) Gene expression of NAD(P)H dehydrogenase [quinone] 1 (NQO1) (each value corresponds to the mean ± SEM of three-fold independently performed experiments). (C) Gene expression of serine palmitoyltransferase 1 (SPTLC1), sphingolipid delta(4)-desaturase DES1 (DEGS1), ceramide synthase 2 (CERS2), and ceramide synthase 6 (CERS6) (each value corresponds to the mean ± SEM of three-fold independently performed experiments). Statistics: Paired Student’s t-test, p>0.05 for all genes evaluated. (DOC) [file pone.0200739.s005.doc]

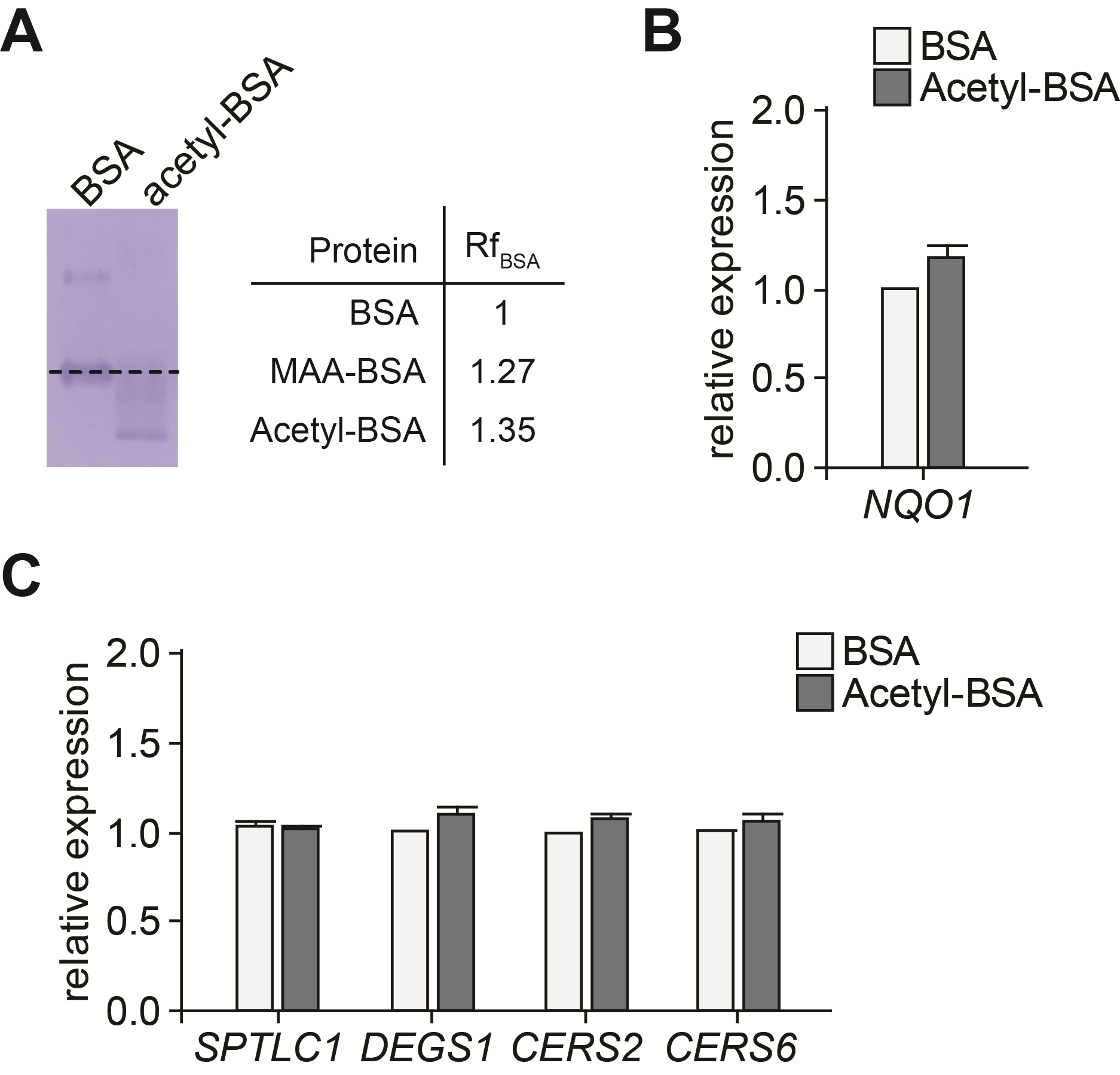


**S2 Fig.** **Influence of acetylated-BSA (acetyl-BSA) in gene expression of WERI-Rb1 cells. (A)** Native PAGE of bovine serum albumin (BSA) and BSA treated with 2 μL of acetic anhydride per mg of protein to produce acetyl-BSA. The gel was stained with Coomassie Blue. The table shows the retention factor relative to BSA (Rf_BSA_) calculated as the ratio of the distance each protein has travelled over the distance travelled by BSA. Rf_BSA_ for MAA-BSA was obtained from the gel showed in S1 Fig. **(B-C)** WERI-Rb1 cells treated with 80 μg/mL of BSA or acetyl-BSA for 24 h. **(B)** Gene expression of NAD(P)H dehydrogenase [quinone] 1 (*NQO1*) (each value corresponds to the mean ± SEM of three-fold independently performed experiments). **(C)** Gene expression of serine palmitoyltransferase 1 (*SPTLC1*), sphingolipid delta(4)-desaturase DES1 (*DEGS1*), ceramide synthase 2 (*CERS2*), and ceramide synthase 6 (*CERS6*) (each value corresponds to the mean ± SEM of three-fold independently performed experiments). Statistics: Paired Student’s *t*-test, p>0.05 for all genes evaluated.
