# Supplementary material for: Evaluation of serum sphingolipids and the influence of genetic risk factors in age-related macular degeneration
Source: PLoS One. 2018 Aug 2;13(8):e0200739. doi: 10.1371/journal.pone.0200739 (PMC6071970; doi:10.1371/journal.pone.0200739)
Supplement: S3 Fig — Expression of 15 genes from the ceramide metabolism evaluated by RT-PCR in retinoblastoma cell line WERI-Rb1. CERS3 presented a very low expression and was not included in later experiments. Ctrl lanes for each gene represent a control without cDNA addition. The lower molecular weight band in UGCG and Ctrl UGCG lanes correspond to primer dimers. (DOC) [file pone.0200739.s006.doc]

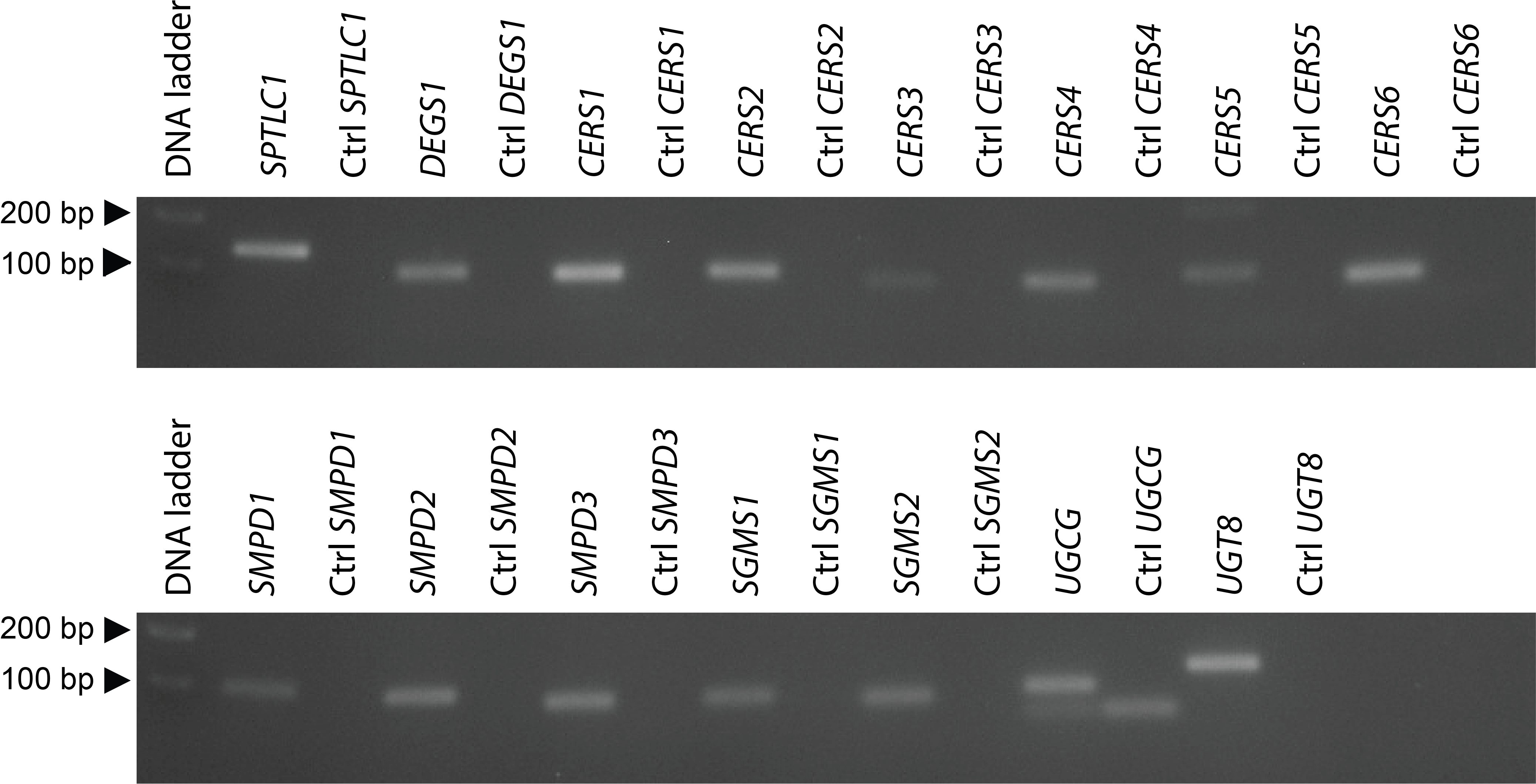


**S3 Fig. Expression of selected genes in the ceramide metabolism in WERI-Rb1 cells.** Expression of 15 genes from the ceramide metabolism evaluated by RT-PCR in retinoblastoma cell line WERI-Rb1. *CERS3* presented a very low expression and was not included in later experiments. Ctrl lanes for each gene represent a control without cDNA addition. The lower molecular weight band in *UGCG* and Ctrl *UGCG* lanes correspond to primer dimers.
